# Setting health research priorities using the CHNRI method: VI. Quantitative properties of human collective opinion

**DOI:** 10.7189/jogh.06.010503

**Published:** 2016-06

**Authors:** Sachiyo Yoshida, Igor Rudan, Simon Cousens

**Affiliations:** 1 Department for Maternal, Newborn, Child and Adolescent Health, World Health Organization, Geneva, Switzerland; 2 Centre for Global Health Research, The Usher Institute for Population Health Sciences and Informatics, The University of Edinburgh, Edinburgh, Scotland, UK; 3 Department of Infectious Disease Epidemiology, London School of Hygiene and Tropical Medicine, London, UK

## Abstract

**Introduction:**

Crowdsourcing has become an increasingly important tool to address many problems – from government elections in democracies, stock market prices, to modern online tools such as TripAdvisor or Internet Movie Database (IMDB). The CHNRI method (the acronym for the Child Health and Nutrition Research Initiative) for setting health research priorities has crowdsourcing as the major component, which it uses to generate, assess and prioritize between many competing health research ideas.

**Methods:**

We conducted a series of analyses using data from a group of 91 scorers to explore the quantitative properties of their collective opinion. We were interested in the stability of their collective opinion as the sample size increases from 15 to 90. From a pool of 91 scorers who took part in a previous CHNRI exercise, we used sampling with replacement to generate multiple random samples of different size. First, for each sample generated, we identified the top 20 ranked research ideas, among 205 that were proposed and scored, and calculated the concordance with the ranking generated by the 91 original scorers. Second, we used rank correlation coefficients to compare the ranks assigned to all 205 proposed research ideas when samples of different size are used. We also analysed the original pool of 91 scorers to to look for evidence of scoring variations based on scorers' characteristics.

**Results:**

The sample sizes investigated ranged from 15 to 90. The concordance for the top 20 scored research ideas increased with sample sizes up to about 55 experts. At this point, the median level of concordance stabilized at 15/20 top ranked questions (75%), with the interquartile range also generally stable (14–16). There was little further increase in overlap when the sample size increased from 55 to 90. When analysing the ranking of all 205 ideas, the rank correlation coefficient increased as the sample size increased, with a median correlation of 0.95 reached at the sample size of 45 experts (median of the rank correlation coefficient = 0.95; IQR 0.94–0.96).

**Conclusions:**

Our analyses suggest that the collective opinion of an expert group on a large number of research ideas, expressed through categorical variables (Yes/No/Not Sure/Don't know), stabilises relatively quickly in terms of identifying the ideas that have most support. In the exercise we found a high degree of reproducibility of the identified research priorities was achieved with as few as 45–55 experts.

In 1906, Galton suggested that a group of individuals tend to make better predictions as a collective than any individual. Since then, our understanding of collective decision–making, termed by some as the “Wisdom of Crowds”, has grown considerably [1]. Crowd–sourcing has become an increasingly important human tool to address many problems – from government elections in democracies [2], formation of stock market prices [3], to modern online platforms such as TripAdvisor (to advise on the best hotels and restaurants) [4] or Internet Movie Database (to advise on the best movies, TV shows, etc.) [5], all of which are based on personal opinions of many hundreds or thousands of participants. The CHNRI method (the acronym for: Child Health and Nutrition Research Initiative) also uses crowdsourcing as the major component of the process to set priorities among many competing health research ideas [6,7]. It relies on large groups of scientists who are invited to participate in each exercise. Within the CHNRI process, several dozens (or even hundreds) of scientists are typically invited first to generate, and then to assess many competing health research ideas using a pre–defined set of priority–setting criteria. Their collective optimism towards each research idea with respect to specific criteria is measured and many research ideas are then ranked according to the scores they achieve across all criteria.

However, researchers typically question several concepts in relation to the “validity” of the CHNRI exercises. The first question is fundamental to the entire process, asking the developers of the method to demonstrate convincingly that the opinion of a large expert group is more reliable and trustworthy than the opinion of only one, or a very small number of experts. This question has been addressed in a previous paper in this series [8], which demonstrated that the collective knowledge of a group (rather than opinion) generally outperforms the knowledge of any single individual. While for factual knowledge there is a “gold standard” against which we can compare the response of the collective to that of individuals, for opinions about future outcomes there is no such “gold standard”. Nevertheless, given that individual knowledge, or lack of it, underlies a significant part of individual opinion, and that the same governing principles that make the collective knowledge superior to individual knowledge (described in our previous paper [8]) should also apply to opinion, we consider this question largely addressed. The substantial literature on so–called “prediction markets” provides further evidence of the reliability and effectiveness of collective opinion in comparison to individual opinion in predicting future events [9,10].

The second question concerns the “optimal” sample size of researchers to be invited to conduct a CHNRI exercise. Here, “optimal” refers to a minimum number of experts needed from a larger, global “pool” of experts, in order to reduce the cost and complexity of conducting the exercise while obtaining a replicable collective opinion. The question of the “sufficient” sample size can be investigated by exploring at which point addition of further experts from the larger, global “pool” of experts ceases to influence the outcomes of the CHNRI process. The third question is related to the composition of the sample of experts, and how this composition can potentially affect the final scores. Do the background characteristics of the experts invited to participate affect their collective opinion in such a way that one subgroup of experts would provide systematically different scores for another subgroup?

In this article, we address the latter two questions by exploring some of the quantitative properties of human collective opinion. We study the special case where the collective opinion is based on a set of individual opinions, all of which are expressed in the form of simple categorical variables. These variables relate to the optimism expressed by each participating expert regarding the extent to which each proposed research idea meets the different priority–setting criteria [6,7]. The opinion provided by the participating experts can be expressed as “Yes” (equals 1), “No” (equals 0), “Not sure” (equals 0.5) and “I don't know” (equals blank input), which is the typical input required in the CHNRI method. This special case is of particular interest, because in our previous paper [8] we demonstrated the effectiveness of this method of expressing individual opinion in comparison to other types. Finally, one of the concerns about this way of collecting opinion from groups of experts is the impact of low response rates and subsequent self–selection bias. We will mention this concern here because we find it potentially very important, although it will be difficult to study and we will not attempt to address it in this paper.

## METHODS

In order to answer the latter two questions posed in the introduction, we conducted statistical analyses of the inputs provided by the group of experts who took part in a previous CHNRI exercise. These analyses focused on identifying whether there was a point of “saturation” in collective opinion. “Saturation” here refers to the idea that beyond a certain sample size of experts, adding further experts' opinions does not significantly change the results of the process. To study this, we used the data set with quantitative input from the experts who took part in a CHNRI exercise on newborn health in this series [11], which is freely available as a supplementary online material to the article in question [11]. All input was provided in the form of a simple categorical variable (ie, optimism towards each idea expressed as “Yes” (equals 1), “No” (equals 0), “Not sure” (equals 0.5) and “I don't know” (equals blank input)).

Our analysis strategy involved drawing many random sub–samples, with replacement, from the full sample of 91 expert participants in the CHNRI exercise on newborn health. The experts scored a set of 205 proposed research ideas [11]. Our aim was to identify the minimum sample size of experts required to produce stable results. We used two metrics to assess stability. First, we compared the 20 most highly ranked ideas for each resampled data set with the 20 most highly ranked ideas in the whole data set (ie, all 91 experts) and calculated how many ideas appeared in both top 20 lists. If all the opinions were assigned entirely at random, then we would only expect about 2 research ideas on average (out of the total of 205) to be in common across two samples. Given this reasonably low expected agreement by chance, we arbitrarily defined results as being stable when 15 (or more) of the 20 highest ranked ideas were concordant with those based on the opinion of the full sample of 91 experts. We believe that such an occurrence indicates a high level of stability/replicability compared with the 2 expected purely by chance.

### Previous studies into the point of saturation in collective opinion

The question of the sample size at which the “saturation” of information occurs has been vigorously discussed over many years in relation to qualitative research, where interviews conducted with the participants are recorded and analysed to obtain insights into a wide variety of research topics. In qualitative research, saturation is typically described in the context of obtaining the “appropriate” sample size at which no new ideas, findings, or problems are found. Determining the “appropriate” sample size is critical, because a sample that is larger than needed would result in inefficient use of research funds, resources and time. On the other hand, too small a sample size may result in limited validity of the research findings.

The idea of “saturation” was first introduced in the late 1960s [12] through the notion that, though every research participant can have diverse ideas in principle, the majority of qualitative studies will inevitably reach a point of saturation. Since the work by Glaser and Strauss [12], researchers have attempted to provide sample size guidance for various research disciplines. Proposed sample sizes have ranged from fifteen in all qualitative research disciplines [13] to sixty [14] in the area of ethnographic interviews. These proposed sample sizes were rarely accompanied by a clear justification or description of how they were derived.

However, the idea of saturation does not necessarily translate to CHNRI exercises, where opinions are submitted in a form of quantitative categorical variables. This gives us perhaps a rare opportunity to perform an assessment of the quantitative properties of human collective opinion by analysing a data set underlying a typical CHNRI exercise. We found one study that attempted to analyse the stability of responses of the 23 health care and patient safety experts who participated in a Delphi survey using a categorical rating scale [15], which is the most similar case to the CHNRI process that we were able to find in the literature. In that study [15], the responses to each item were scored on a rating scale from 1 to 4, with “1” being unimportant to “4” being very important. The responses obtained in the first round of the survey were processed using sampling with replacement to produce hypothetical samples of 1000 and 2000 participants, from the initial sample size of 23 subjects. Then, means and 95% confidence intervals for the scores of the original 23 participants were compared with the hypothetical samples. Substantial similarity of inferential statistics between the actual and hypothetical samples was observed, from which the authors concluded that the “stability” of results was already achieved with only 23 actual study participants [15]. Clearly, this interpretation was limited by having an original sample as small as 23 individuals to generate large bootstrapping samples, and the result needs to be replicated using a larger initial sample of individuals to generate bootstrapping samples. In our study, the key improvement will be drawing sub–samples smaller than the original sample, while in the approach described in this study samples were created that were much larger than the original sample – which is an approach with major limitations.

### Defining “saturation” in our study

In our study, we defined “saturation” in two ways. First, we defined it as the point where we observed replicability in the collective rankings of top 20 research ideas (among a total of 205 assessed) between two randomly generated sub–samples of a given sample size. In other words, involving further experts would no longer be expected to make any important difference to the 20 most highly ranked priorities. Given that randomness inherent to the process of sampling makes it unrealistic to expect all 20 priorities to always replicate at a certain sample size, and taking into account low “a priori” probability of replication (only 2 among the 20 most highly ranked research ideas would be expected to replicate by chance alone), we needed to define “saturation” arbitrarily. We considered the specific sample size as “saturation–reaching” when the same 15 (or more) research ideas in any two randomly generated samples of a specific size were expected to be found among the 20 most highly ranked research ideas in both samples.

Second, we used Spearman’s rank correlation coefficient to compare the ranks assigned to all 205 proposed research ideas by the randomly generated sub–samples with the ranks derived from the full sample. We considered “saturation” to be achieved when the median rank correlation coefficient reached or exceeded 0.95 (which is an extremely high rank correlation coefficient). We believe that both definitions of saturation are stringent and conservative from the statistical point of view.

### Database used in this analysis

We used anonymised raw scores provided by the participants in the CHNRI exercise on newborn health [11]. The database included all individual scores from 91 participating experts that were assigned to all 205 proposed research ideas using 5 pre–defined criteria. The criteria used in the exercise are summarized in **Box 1**, and they were posed in the form of simple “yes/no” questions. The requested input was provided in the form of numbers: 0 (meaning “no”), 0.5 (“informed, but undecided answer”), 1 (“yes”), and blank (“insufficiently informed”). “Blank” was used whenever the participants did not feel that they possessed enough technical knowledge to be able to answer, which is different from an “informed, but undecided” answer, where the expert could neither agree nor disagree although they felt that they had enough knowledge on the topic.

Box 1 **The five criteria used in the exercise.**

Criterion 1. Answerability: Can the research question be answered ethically?

Criteria 2. Efficacy/Effectiveness: Can the new knowledge lead to an efficacious intervention or programme?

Criteria 3. Deliverability and acceptability: Is the proposed intervention or programme deliverable and acceptable?

Criteria 4. Maximum potential for disease burden reduction: Can the intervention or program improve newborn health substantially?

Criteria 5. Effect on equity: Can the interventions on program reach the most vulnerable groups?

### Statistical analysis

We used resampling with replacement, sometimes referred to as “bootstrapping”, to simulate the diversity of samples drawn from a larger global pool of experts. All analyses were performed using the statistical program STATA 13.0 (www.stata.com). To study how the rankings assigned to proposed research ideas change and converge with increasing sample sizes of experts, we generated samples ranging in size from minimum 15 to a maximum of 90. For each selected sample size, 1000 random bootstrap samples were drawn.

Two statistical analyses were then performed to examine how the ranking list of research ideas changed as the number of experts contributing to the CHNRI exercise increased. In the first analysis, we examined the concordance in the top 20 research ideas between 1000 randomly generated subsamples of the same size that were developed using the bootstrap method. In the second analysis, we used Spearman's rank correlation coefficient to examine the concordance in the ranking order of all 205 research ideas between 1000 randomly generated subsamples of the same size that were developed using the bootstrap method.

### Analysis of subgroups within the full sample

Research priority scores (RPS) were recalculated for each research question in sub–samples of scorers that were defined by participants’ self–classified background and the country in which they were based. Participants originally classified themselves as researchers, policy makers, donor representatives, program managers or health practitioners (multiple choices were not allowed), and this information is available in the original paper [11]. In this exercise, we had combined all categories other than researcher into one category as “non–researcher”, as the numbers of participants falling into each of the non–researcher categories were small. The country where the scorer was based was classified by the level of income as either a “high–income country” (HIC) or a “low– or middle–income country” (LMIC), using the World Bank's categorization [16]. We explored: (i) the differences in median scores that different sub–groups of scorers (ie, researchers vs non–researchers; and HIC–based vs LMIC–based) assigned to different criteria; the median scores were determined across all 205 research ideas to investigate whether subgroups of scorers systematically scored particular criteria differently; (ii) the overlap between the top 20 research ideas identified by different sub–groups of scorers (ie, researchers vs non–researchers; and HIC–based vs LMIC–based).

## RESULTS

**Figure 1:**
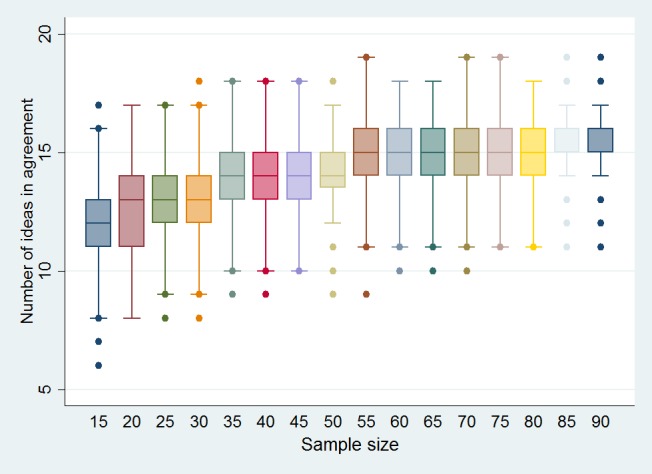
Level of overlap among the top 20 ranked research ideas (Y–axis) by the size of the sample of randomly selected experts (X–axis) from a total pool of 91 experts using a bootstrap method (simulation 1000 times with replacement of already selected experts, using bsampling function). The size of randomly generated samples ranged from 15 to 90 and it was based on the CHNRI exercise on newborn health research priorities [11].

**Figure 1** shows the how concordance with respect to the top 20 priorities increased as the number of sampled scorers increased. Note that when resampling 90 scorers with replacement, concordance with the top 20 priorities based on the original sample of 91 experts would not be expected to reach 100%. This reflects the fact scores derived from the original sample of 91 experts are themselves subject to sampling variation. The median concordance (across the 1000 sub–samples drawn for each sample size) increases from 12/20 (60%) with a sample size of 15 to 15/20 (75%) with a sample size of 55 experts. Thereafter there is no clear improvement in concordance with increasing sample size. The interquartile range for concordance with a sample size of 55 is 14/20 to 16/20 (70% to 80%) and this also appeared relatively stable as sample sizes were increased further. At a sample size of 90, the median concordance was 16/20 (85%) (IQR 15–16). Given that this gives an indication of the variability of the sample size we had available to us for analysis, it appears that relatively stable results can be achieved with sample of 50 experts (median 14, IQR 13.5–15). There is little further increase in achieved overlap by increasing the pool of experts from 50 to 90 (**Figure 1**).

**Figure 2:**
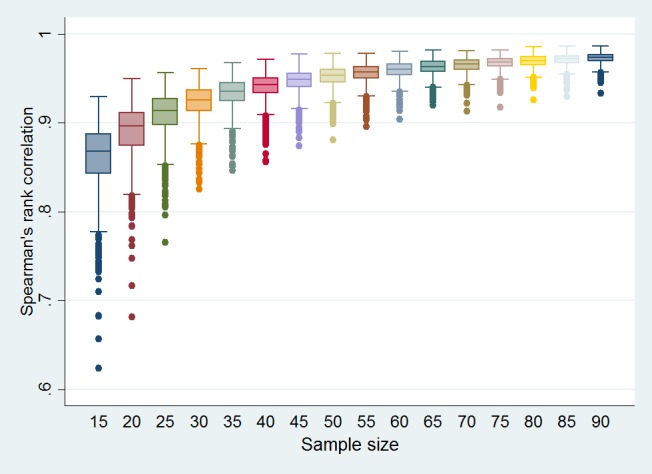
Spearman’s rank correlation among all 205 ranked research ideas (Y–axis) by the size of the sample of randomly selected experts (X–axis) from a total pool of 91 experts using a bootstrap method (simulation 1000 times with replacement of already selected experts, using bsampling function). The size of randomly generated samples ranged from 15 to 90 and it was based on the CHNRI exercise on newborn health research priorities [11].

**Figure 2** shows the relationship between the sample size of the scorers within the CHNRI newborn health exercise [11] and the median, IQR and range of Spearman’s rank correlation for the ranks of all 205 proposed research ideas. As expected, the rank correlation coefficient increases as sample size becomes larger and a median correlation of 0.95 was reached at the sample size of 45 experts (median of the rank correlation coefficient = 0.95; IQR 0.94–0.96).

Among the 91 scorers in the newborn health exercise, 61 self–classified as “researchers” and 30 as “non–researchers”; 53 participants were based in HIC and 38 in LMIC. **Table 1** shows the differences in median scores (with inter–quartile range, IQR) that different subgroups of scorers (ie, researchers vs “non–researchers”; and high–income country (HIC)–based vs low– or middle–income country (LMIC)–based) assigned to different criteria. The differences between researchers and non–researchers were small, with non–researchers being slightly more optimistic about maximum potential impact, but all differences were well within the limits predicted by inter–quartile ranges. Larger differences were observed between HIC–based and LMIC–based researchers, with the latter tending to provide more optimistic scores, ranging from a 7 to a 24 point–difference on a scale from 0 to 100. The smallest difference was noted for answerability, followed by effectiveness and deliverability, while the largest differences were noted for maximum potential impact and equity.

**Table 1:** The differences in median scores (with inter–quartile range, IQR) that different sub–groups of scorers (ie, researchers vs “non–researchers”; and high–income country (HIC)–based vs low– or middle–income country (LMIC–based) assigned to different criteria*

	All scorers (median, IQR) (N = 91)	Researchers (median, IQR) (N = 61)	“Non–researchers” (N = 30)	HIC–based (median, IQR) (n = 53)	LMIC–based (median, IQR) (n = 38)
Total score	63 (54–71)	62 (54–70)	64 (53–73)	57 (47–66)	72 (61–80)
Answerability	76 (68–83)	76 (68–84)	77 (67–85)	74 (63–81)	81 (73–89)
Effectiveness	70 (61–77)	69 (61–78)	68 (59–78)	66 (54–74)	76 (66–84)
Deliverability	69 (58–77)	69 (59–78)	67 (57–78)	65 (54–72)	77 (65–84)
Maximum impact	42 (32–52)	39 (32–50)	44 (32–55)	32 (23–41)	54 (44–66)
Equity	57 (47–70)	57 (46–66)	60 (46–75)	48 (37–61)	72 (60–81)

IQR – interquartile range, HIC – high–income, LMIC – low– and middle–income

*The median scores were determined across all 205 research ideas in order to investigate if any sub–group of scorers deviated in their scoring of any particular criterion.

**Table 2** shows the overlap between the top 20 research ideas (RI–) identified by different sub–groups of scorers (ie, researchers vs “non–researchers”; and HIC–based vs LMIC–based). There was an overlap between researchers and “non–researchers” for 10 out of top 20 research ideas (50%). For HIC–based vs LMIC–based researchers, 8 of top 20 research ideas (40%) overlapped. We could judge this level of overlap against the expectation provided by the bootstrap analysis for comparable sample sizes. There is likely to be an effect of sub–stratification, which is smaller for the “researchers vs. non–researchers” comparison, but more considerable for the “HIC–based vs. LMIC–based” comparison.

**Table 2:** The overlap between the top 20 research ideas (RI–) identified by different sub–groups of scorers (ie, researchers vs “non–researchers”; and HIC–based vs LMIC–based)*

Rank	All scorers (n = 91)	Researchers (n = 60)	“Non–researchers” (n = 31)	HIC–based (n = 53)	LMIC–based (n = 38)
1	RI–30	**RI–30**	**RI–30**	**RI–30**	**RI–30**
2	RI–28	**RI–28**	**RI–28**	**RI–28**	**RI–23**
3	RI–15	**RI–15**	**RI–15**	RI–29	**RI–15**
4	RI–23	RI–29	RI–5	**RI–15**	RI–47
5	RI–33	**RI–23**	**RI–33**	**RI–33**	**RI–28**
6	RI–29	RI–36	RI–79	RI–7	RI–44
7	RI–149	RI–7	**RI–23**	RI–13	RI–18
8	RI–37	RI–13	RI–52	**RI–23**	RI–12
9	RI–5	**RI–33**	**RI–149**	RI–149	**RI–33**
10	RI–13	RI–58	RI–46	RI–36	RI–86
11	RI–79	**RI–149**	RI–47	RI–5	RI–58
12	RI–78	**RI–37**	RI–44	RI–37	**RI–46**
13	RI–36	RI–67	**RI–8**	RI–21	RI–60
14	RI–46	RI–75	**RI–78**	RI–55	RI–11
15	RI–8	**RI–78**	RI–129	**RI–79**	RI–8
16	RI–55	RI–86	RI–11	RI–22	RI–35
17	RI–52	**RI–55**	**RI–37**	RI–52	RI–67
18	RI–75	RI–12	**RI–55**	**RI–78**	RI–10
19	RI–58	**RI–8**	RI–127	RI–75	**RI–79**
20	RI–67	RI–158	RI–138	**RI–46**	**RI–78**

HIC – high–income, LMIC – low– and middle–income

*The research ideas that overlap between researchers vs “non–researchers”, and HIC–based vs LMIC–based sub–samples, respectively, are in bold for easier recognition. Note: eg, RI–30 indicates research idea number 30 in the list of 205 ideas.

## DISCUSSION

In this paper, we addressed two important questions relating to the quantitative properties of human collective opinion: (i) whether there is a point of “saturation” in the sample size, after which no significant changes in the collective opinion should be expected when more experts are brought into the exercise; and (ii) whether there is evidence that opinions differ between subgroups of experts defined by their professional background or their geographic location. We addressed both questions using data from a previous CHNRI exercise [11]. The data set based on the CHNRI exercise was useful in this regard, because it quantified a large number of expert opinions about 205 competing research ideas in a systematic and structured way, based on five pre–defined criteria, using simple categorical responses. We did not attempt to demonstrate that the collective would give more “useful” predictions than individual experts would, since this is examined in another paper on collective knowledge [8]. Perhaps the best support for the view that the opinion of a collective will prove more useful over time than that of individuals is provided in the literature on stock markets and prediction markets [3,9,10]. Over long periods of time, following the collective wisdom seems to be the most successful strategy. There are some important differences, though, because stock markets to a degree involve betting individual opinions against those of others, where investors are trying to identify stocks and shares that are undervalued by the collective opinion. Together, our previous paper from this series [8] and the large experience with stock markets and prediction markets [3,9,10] make a compelling case for collective decision–making.

Our analyses indicate that, in bootstrap samples that ranged in size from only 15 to 90, the level of overlap among the top 20 scored research ideas increased with sample size up to about 50–55 experts. At this point, the median level of concordance stabilized at 15/20 top ranked questions (75%), with the interquartile range also generally stable (14–16). There was little further increase in overlap when the bootstrap sample of experts increased from 55 to 90. However, it should be noted that the overlap of 12/20 top ranked research ideas was achieved with sample sizes as small as 15 experts, as opposed to only 2 research ideas that would have been expected by chance. The conclusion from this analysis is that human collective opinion, when expressed in simple quantitative terms, tends to converge towards a similar outcome and saturate quickly. A sample size of 15 persons already shows an appreciable level of reproducibility, but with 50–55 experts the level of replicability becomes nearly equal to that which is achievable with a sample size of 90..

It is important to note that the total sample of 91 experts, which is the maximum that we had available, represents only a sub–sample of a much larger global pool of experts. Therefore, it also carries a certain inherent random variation relative to the “total expert population”. Sampling with replacement enables us to examine how variable the results for a given sample size will be, assuming that are full sample of 91 experts is representative of the diversity of the wider global pool. Thus two bootstrapped samples of size 91participants would not be expected to have the top 20 research ideas fully replicated (although this is the entire original sample!). We used sampling with replacement to overcome, at least partly, the concern that the 91 experts are still only a reasonably small sample of the larger population and to produce a conservative estimate of the minimum sample size that that produces replicable results in this particular CHNRI exercise.

We also tested the relationship between the sample size of the scorers and Spearman’s rank correlation coefficient for the ranks of all 205 proposed research ideas. As expected, the rank correlation coefficient increased as the bootstrap sub–samples became larger. A median correlation of 0.95 was reached at the sample size of 45 experts (median of the rank correlation coefficient = 0.95; IQR 0.94–0.96), which again points to high reproducibility and relatively quick saturation.

Studying quantitative properties of human collective opinion, as opposed to collective knowledge verifiable against accepted facts, has the limitation that no gold standard is available against which the “accuracy” of the opinion can be judged. We therefore focused on the questions of saturation, reproducibility and subgroup stratification. Another limitation of this preliminary analysis is that it was based on a single data set from a previous CHNRI exercise. An analysis of multiple data sets with large numbers of experts and different numbers of research ideas being scored may offer further interesting insights into a nature of human collective opinion and results that are more generalizable than those based on the analysis of a single data set. Ideally, an analysis should involve as many experts as possible, because testing on exercises that only included reasonably small groups of experts will not be very useful. At this point, we should also declare that we can't predict the effects of low response rate and self–selection bias on the level of saturation achieved. The issue of missing responses of the experts who do not choose to participate should be explored separately and it remains an unresolved uncertainty related to the validity of the approach used in the CHNRI method.

Any future work in this area could plan to acquire more data sets and replicate the analyses from this study. One emerging question that it would be interesting to answer is to examine the main determinants of the observed level of concordance in ranking lists. Examples of possible determinants are the composition and the nature of the proposed research ideas, the composition and sample size of scorers, and the criteria used for discrimination. Answering this question would require a study into how an increasing number of experts participating in the CHNRI exercise introduces variation in the data set across different exercises; then, how does the number of research questions in the data set introduce variation; how does the substance (ie, content, plausibility) of research ideas introduce variation; and how does the level of agreement between all experts participating in the CHNRI exercise introduce further variation. It would be important to understand whether the key determinant of variation in the data set is the number of experts, the diversity of experts, the number of research ideas, or the content and diversity of research ideas. This could be understood if the number of research ideas and the number of experts are standardized (ie, made equal) across several different CHNRI exercises and then the rank correlation analysis and a comparison of the concordance of the top 20 research priorities are repeated using the methodology in this paper.

An important question is whether by increasing the sample size of scorers we would obtain a wider spectrum of opinions, and therefore greater variation between responses, or whether we would simply continue to observe the same level of variation. One way of addressing this would be to look at a CHNRI exercise where we could separate those who responded to the initial request and those who only responded after reminders, and study whether there was evidence that the late responders differed from the early responders in their opinions.

A search for the presence of sub–stratification in this study could only examine the two characteristics that were known for each invited scorer: a background in research vs “non–researchers”, and affiliation to HIC vs LMIC. When the analysis of concordance was conducted, a reduced level of agreement was detectable when HIC–based vs LMIC–based samples were compared. This observation lends support to the recommendation that an inclusive approach to the sample selection in the CHNRI method should be preferred, so that the result of the exercise reflects the opinion of a wide group of experts. This should help to prevent any particular sub–group among the scorers, with particular views, having undue influence on the results. An analysis of a much larger set of data set from the CHNRI exercises might help to suggest how best to manage the problem of sub–stratification within the sample of invited experts and whether there were examples of exercises in which this concern was reduced to a minimum, or even avoided [17].

Finally, it is of interest to the field of qualitative research to draw analogies between the observations on “saturation” of quantitatively expressed human collective opinion, which we observed in this study, and the long–term notion of quick saturation of information content obtained through interviews with human subjects. Researchers studying the question of the “saturation of ideas” in qualitative research often conclude that 15 interviews may be all it takes to reach a very high degree of “saturation”, with 20–30 interviews being sufficient [18]. The numbers as small as those proposed are often counter–intuitive to researchers who conduct quantitative research in the fields such as epidemiology, public health and/or clinical trials, where new information is still discovered even after hundreds or thousands of participants have been enrolled, and having larger sample sizes often leads to a better study with more statistical power to demonstrate convincing results. We conclude that the results of our study seem to support the notion that human collective opinion tends to saturate surprisingly quickly and there does seem to be a point at which adding further experts is unlikely to significantly affect the results that were derived from the initial 45–55 experts. This interesting finding warrants further exploration to understand why this seems to be the case and whether there is a wider significance of this finding, or perhaps any immediate opportunities to implement it in solving practical problems in different areas of human activity.
